# Repair of bone defects in rhesus monkeys with α1,3-galactosyltransferase-knockout pig cancellous bone

**DOI:** 10.3389/fbioe.2022.990769

**Published:** 2022-09-12

**Authors:** Wenhao Wang, Jiansen Lu, Ying Song, Chun Zeng, Yongkui Wang, Cheng Yang, Bin Huang, Yifan Dai, Jian Yang, Liangxue Lai, Liping Wang, Daozhang Cai, Xiaochun Bai

**Affiliations:** ^1^ Department of Orthopaedics, Shandong Provincial Hospital Affliated to Shandong First Medical University, Jinan, China; ^2^ Academy of Orthopedics, Guangdong Province, Department of Orthopedics, The Third Affiliated Hospital, Southern Medical University, Guangzhou, Guangdong, China; ^3^ Department of Joint Surgery, The Fifth Affiliated Hospital of Southern Medical University, Guangzhou, Guangdong, China; ^4^ Department of Immunology, School of Basic Medical Sciences, Southern Medical University, Guangzhou, Guangdong, China; ^5^ Department of Endodontics, Jinan Stomatological Hospital, Jinan, China; ^6^ State Key Laboratory of Reproductive Medicine, Jiangsu Key Laboratory of Xenotransplantation, Nanjing Medical University, Nanjing, Jiangsu, China; ^7^ Department of Biomedical Engineering, Materials Research Institute, The Huck Institutes of the Life Sciences, The Pennsylvania State University, University Park, PA, United States; ^8^ Key Laboratory of Regenerative Biology, South China Institute for Stem Cell Biology and Regenerative Medicine, Guangzhou Institutes of Biomedicine and Health, Chinese Academy of Sciences, Guangzhou, Guangdong, China; ^9^ UniSA Clinical and Health Sciences, University of South Australia, Adelaide, SA, Australia; ^10^ Department of Cell Biology, School of Basic Medical Science, Southern Medical University, Guangzhou, China

**Keywords:** cylindrical bone defect, gene knockout pig, xenotransplantation, bone transplantation, repair

## Abstract

**Introduction:** Since xenografts offer a wide range of incomparable advantages, they can be a better option than allografts but only if the possibility of immunological rejection can be eliminated. In this study, we investigated the ability of α1,3-galactosyltransferase (α1,3-GT) gene knockout (GTKO) pig cancellous bone to promote the repair of a femoral condyle bone defect and its influence on heterologous immune rejection.

**Materials and methods:** Cylindrical bone defects created in a rhesus monkey model were transplanted with GTKO bone, WT bone or left empty. For immunological evaluation, T lymphocyte subsets CD4^+^ and CD8^+^ in peripheral blood were assayed by flow cytometry, and the IL-2 and IFN-γ contents of peripheral blood serum were analyzed by ELISA at 2, 5, 7, 10, and 14 days post-surgery. Micro-CT scans and histological assessment were conducted at 4 and 8 weeks after implantation.

**Results:** Compared with WT-pig bone, the heterologous immunogenicity of GTKO-pig bone was reduced. The defect filled with fresh GTKO-pig bone was tightly integrated with the graft. Histological analysis showed that GTKO-pig cancellous bone showed better osseointegration and an appropriate rate of resorption. Osteoblast phenotype progression in the GTKO group was not affected, which revealed that GTKO-pig bone could not only fill and maintain the bone defect, but also promote new bone formation.

**Conclusion:** GTKO-pig cancellous bone decreased the ratio of CD4^+^ to CD8^+^ T cells and cytokines (IFN-γ and IL-2) to inhibit xenotransplant rejection. Moreover, GTKO group increased more bone formation by micro-CT analysis and osteoblastic markers (Runx2, OSX and OCN). Together, GTKO-pig cancellous bone showed better bone repair than WT-pig cancellous bone.

## Introduction

The optimal treatment of large bone defects still poses a serious challenge in reconstructive orthopedic, craniofacial, oral, and plastic surgery procedures. The primary method of repairing bone defects is bone transplantation, which includes the use of autologous bone grafts, bone allografts, and xenografts (Trabuco et al., 2007; Li et al., 2015; Beer et al., 2019; Chernchujit et al., 2019). A cancellous bone autograft stimulates the appearance of osteogenic cells. These form new bone from fibrous tissue and they also arrange themselves along the transplanted bone trabeculae creating new bone in this area (Bruder et al., 1994; Aspenberg et al., 1996). Due to its osteoconductive and osteoinductive properties, autologous cancellous bone remains the gold standard for bone grafting procedures. Autologous cancellous bone is usually taken from cancellous bone of the iliac crest, the distal femur, the greater trochanter, or the proximal tibia (Oryan et al., 2014). In addition to its limitation with regard to bone volume, the use of autologous cancellous bone is associated with various complications such as increased trauma, blood loss, pain in the donor site, and prolonged recovery times. In addition, critical size bone defect healing always requires special bone graft materials and the use of bone autografts in osteoporotic populations is associated with a significant reduction in bone quality and quantity, which may contraindicate its use (Sun et al., 2014; Walsh et al., 2017; Chao et al., 2021).

Consequently, allografts have emerged as an alternative source of autologous bone and are widely used despite their inferior osteogenic properties (Park et al., 2014). However, the disadvantages of allografts such as the increased risk of disease transmission and ethical issues have led to the adoption of novel bone graft substitutes. With the shortage of donors for transplantation, xenotransplantation has emerged as an alternative option (Yang et al., 2020).

In view of their size, breeding characteristics, and similarity of organ systems to those of humans, pigs are considered to be the preferred donor for xenotransplantation (Sachs, 1994; Zeyland et al., 2015). Although the feasibility of xenotransplantation has increased as a result of immunological developments, hyperacute rejection (HAR) remains the major barrier to xenotransplantation from pig to human. Galactose-α1,3-galactose (α1,3-Gal) epitopes are a common carbohydrate structure on the cell surface of almost all mammals with the exception of humans and non-human primates (NHP) (Dai et al., 2002; Butler et al., 2016; Zhang et al., 2017). The immune system in humans and NHPs reacts against α1,3-Gal epitopes on xenografts in a T cell-dependent response (Tanemura et al., 2000) and induces sustained inflammation (Feng et al., 2006). The α1,3-galactosyltransferase (α1,3-GT) gene knockout (GTKO) pigs, who show no α1,3-Gal antigen on the surface of cells and tissues, have made it possible for surgeons to resolve the major cause of HAR in xenotransplantation from pig to human (Phelps et al., 2003). Since heterologous bone grafts are free transplants, the severity of HAR is relatively low compared to other solid organ transplantation. However, due to the presence of bone microvascular endothelial cells and the cell immune response mediated by T lymphocytes, heterologous bone grafts will still suffer from HAR, acute immune rejection and chronic immune rejection (Yu et al., 2015). The binding reaction of α1,3-Gal epitopes and natural antibodies is the major cause of HAR in pig-to-human xenotransplantation. Using the organs of GTKO pigs avoids hyperacute rejection leading to increased xenograft survival in NHPs (Kuwaki et al., 2005; Yamada et al., 2005).

**SCHEME 1:**
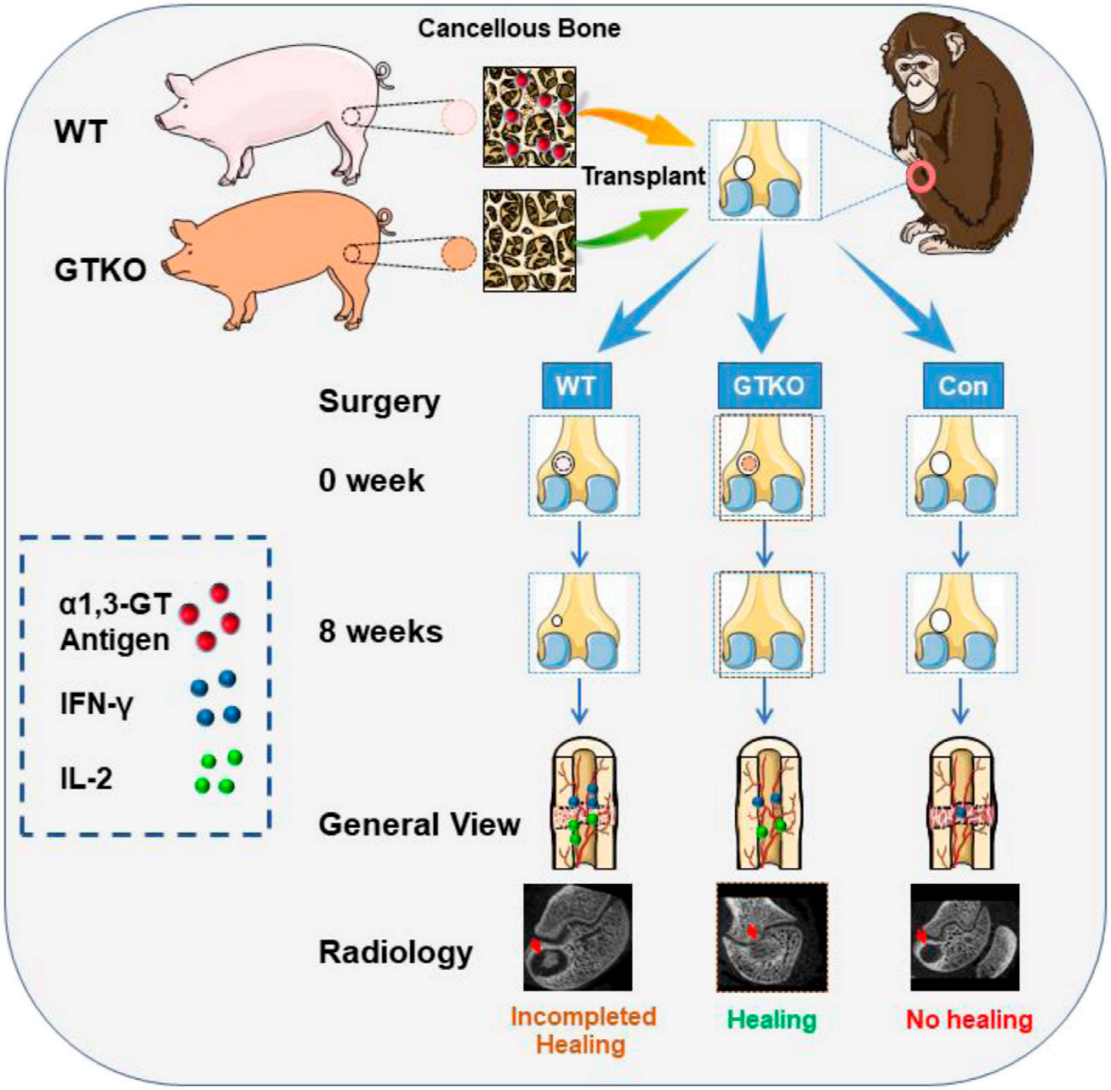
The illustration of GTKO-pig cancellous bone promoting bone defect. WT-pig and GTKO-pig cancellous bone were transplanted for the repair of femoral condyle bone defect cavities in rhesus monkeys. GTKO-pig cancellous bone decreased xenotransplant rejection by reducing the cytokines IFN-γ and IL-2 release and promoted new bone formation by enhancing osteoblast differentiation.

Similar to humans, rhesus monkeys do not have a functional copy of the α1,3-GT gene, so there is no α1,3-Gal expression on the cell surface. In our study, we trimmed GTKO-pig cancellous bone into xenograft cancellous bone plugs, and wild-type pig cancellous bone was used as control grafts for the repair of femoral condyle bone defect cavities in rhesus monkeys. Using this model, the biocompatibility, osteoconductivity, osteoinductivity and heterologous immune rejection of GTKO-pig cancellous bone after xenotransplantation can be precisely assessed and analyzed *in vivo*. Interestingly, GTKO-pig cancellous bone not only decreases xenotransplant rejection, but also promotes new bone formation within the defects (Scheme 1). Moreover, the effects of GTKO-pig cancellous bone in facilitating bone defect healing and its potential and prospects for use in clinical application of bone repair can be ultimately evaluated.

## Materials and methods

### Animals

Chinese Wuzhishan minipigs were used in this study. Transgenic α-1,3-galactosyl transferase nullizygous (GTKO) pigs (*n* = 4) or their hemizygous littermates (phenotypic WT) (*n* = 4) were used as donors. All pigs were male, over 1-year-old, and weighing 70 kg, which were kindly donated by Liangxue Lai and were bred in the Key Laboratory of Regenerative Biology at South China Institute for Stem Cell Biology and Regenerative Medicine (Lai et al., 2002). Healthy and outbred adult male rhesus monkeys were obtained from the Guangdong Landau Biotechnology Co. Ltd., China. Eighteen male animals, aged 3 years with a mean body weight of 3.6 kg, were used in this study. All animal care, experimental and surgical processes and postoperative euthanasia comply with the ARRIVE guidelines and were performed in strict accordance with the ethical principles of the NIH Guide for the Care and Use of Laboratory Animals (NIH Publications No. 8023, revised 1978), after approval by the Institutional Animal Care and Use Committee at Guangdong Landau Biotechnology Co., Ltd. (No. LD20141115). All efforts were made to minimize the animal suffering and the number of animals used. Animals were housed one per cage and provided free access to food and water throughout the study; they were assessed for tuberculosis, SIV, herpes A and B virus.

### Surgical procedure

All animal experiments using rhesus monkeys were approved by Southern Medical University’s Animal Care and Use Committee (Guangzhou, China) and were performed in accordance with the relevant ethical regulations. The animals were randomly divided into three groups: GTKO-pig cancellous bone group, wild-type-pig cancellous bone group, and unrepaired defect (control) group, each containing six monkeys. Before surgeries, the animals received tracheal intubation after intramuscular anesthesia with ketamine and xylazine (Supplementary Figure S1A). All surgeries were performed under aseptic conditions. Right hind limbs were spread by sterile fusion and a 1.5-cm medial incision was made on the lateral knee to expose the lateral femoral condyle. A cylindrical bone defect (diameter 5 mm, depth 8 mm) was produced with a mosaic plasty harvester (Smith & Nephew, Memphis, TN, United States) in the lateral femoral condyles of right knees of each animal (Supplementary Figure S1B). Implants were trimmed to create a bone lock bolt (Supplementary Figure S1C). According to the grouping, implants matching the size of the defect were inserted by press fitting (Supplementary Figure S1D). Three doses of gentamicin (10 mg) were administered to prevent post-operative infection. Animals were ultimately anesthetized and sacrificed by xylazine injection at four or 8 weeks after implantation (Supplementary Figures S1E, 2F). Specimens were fixed in 4% paraformaldehyde, and gross observations were recorded with a digital camera (Supplementary Figures S1G,H).

### Flow cytometry

At 2, 5, 7, 10 and 14 days after operation, 1 ml anticoagulated whole blood was drawn from each monkey. Separate 100-μl aliquots of whole blood were labeled with primary FITC-conjugated antibodies against human CD4 (Biolegend, #344604) and CD8 (Biolegend, #344604), which are known to cross-react with monkey antigens. Flow cytometric (FCM) measurement was performed on a FACS Aria flow cytometer (BD Biosciences, San Jose, CA, United States). FLOW JO software (Treestar Inc., Ashland, OR, United States) was used for data analysis.

### Enzyme linked immunosorbent assay analysis

Monkey IFN-γ (Interferon Gamma) ELISA Kit (Elabscience Biotechnology, Bethesda, MD, United States; #E-EL-MK0002c) and Monkey IL-2 (Interleukin 2) ELISA Kit (Elabscience, #E-EL-MK0006c) were used in our study. The data use Multiple *t* tests.

### Micro-computed tomography analysis

At 4 or 8 weeks after operation, the monkeys were sacrificed and the femoral condyles were dissected, fixed for 48 h in 4% paraformaldehyde and analyzed at 20 μm resolution on a micro-CT Scanner (Viva CT40; Scanco Medical AG, Bassersdorf, Switzerland). We scanned the whole femoral condyle and defined bone tissue around the implants as volume of interest, including the entire trabecular compartment extending 7 mm from the center axis of the implant. The extent of implant bone regeneration was measured by constructing a three-dimensional structure and performing morphometry. Two independent observers made a thorough assessment of the micro-CT scans with axial, coronal, sagittal, and three-dimensional reconstruction of the defects. The micro-CT images were compared between groups at each time point.

To further evaluate bone regeneration at the periphery of the implant areas, we analyzed the trabecular bone volume fraction (BV/TV), trabecular thickness (Tb. Th), trabecular number (Tb. N) and trabecular separation (Tb. Sp) of a hollow cylindrical volume of interest (VOI-I), which was 5.0 mm in external diameter, 4.0 mm in inner diameter, and 800 mm deep (Supplementary Figure S1I).

### Preparation of decalcified sections, histology, immunohistochemistry and histomorphometric assessment

Three animals were sacrificed at 4 and 8 weeks after implantation, and half of the specimens were decalcified and sectioned. The defect and adjacent host bone dissected from the rhesus monkeys were fixed using 4% paraformaldehyde in PBS at 4°C for 48 h and then decalcified in 14% free acid EDTA, pH 7.2, with rocking, changing the solution daily, at 4°C for a minimum of 2 weeks. The specimens were then embedded in paraffin, and longitudinally sectioned at a 2–5 μm thickness with a Leica RM2235 saw microtome (Leica Microsystems Ltd., Wetzlar, Germany) for histological analyses. Hematoxylin and eosin, toluidine blue and Masson’s trichrome staining was performed as previously described (Lin and Hankenson, 2011). Tartrate-resistant acid phosphatase (TRAP) staining was performed according to a standard protocol provided by the supplier (Sigma-Aldrich, St Louis, MO, United States). Four sections from each specimen were scored by two independent blinded observers using Nilsson’s criteria (Hollinger and Kleinschmidt, 1990; Xie et al., 2007).

For IHC, tissue sections were incubated with primary antibodies against osterix (OSX; Abcam, Cambridge, UK; 1:500, ab22552), osteocalcin (OCN; Abcam, 1:500, ab93876), Runx2 (Cell Signaling Technology, Danvers, MA, United States; 1:100), or COL1A1 (Sigma-Aldrich, #E7031-3G3, 1:200 dilution) overnight at 4°C. After washing, the sections were then incubated with secondary antibodies conjugated with HRP at room temperature for 1 h. The DAB chromogenic kit (Boster Biologics, Pleasanton, CA, United States; #AR1020) was used to detect secondary antibodies conjugated with HRP. In immunohistochemistry assays, cells per bone perimeter (B. Pm) was used to calculate the number of positive cells, and integrated optical density per area of positive cells (IOD/area, mean density) was used to quantify the staining intensity by analyzing four different images taken at ×400 magnification with Image Pro Plus 6.0 software (Media Cybernetics, Rockville, MD, United States) (Cooper et al., 1993). An Olympus CX31 microscope was used for imaging and analysis.

### Preparation of undecalcified histological sections

After dissection, the undecalcified bone specimens were fixed in 4% paraformaldehyde for 48 h. Dehydration was achieved by immersing the specimen in serial ethanol solutions from 70% to 100%, before embedding in methylmethacrylate (MMA). Specimens were cleared in xylene, and 5 μm-thick sections were prepared for Goldner’s-Masson trichrome staining (Hering et al., 2006).

### Statistical analysis

IBM SPSS version 20.0 statistical software (IBM, Armonk, NY, United States) was used for analysis and processing. Measurement statistics were described by the mean and standard deviation (mean, SD). Separate effects were analyzed using one-way analysis of variance (ANOVA). The Welch test was conducted if heterogeneity of variance was detected. The LSD method was used when homogeneity of variance was found, while the Games–Howell method was utilized in cases of heterogeneity of variance. * represents *p* value < 0.05 and **p value < 0.01.

## Results

### Immunological assessment of xenotransplant rejection

**FIGURE 1:**
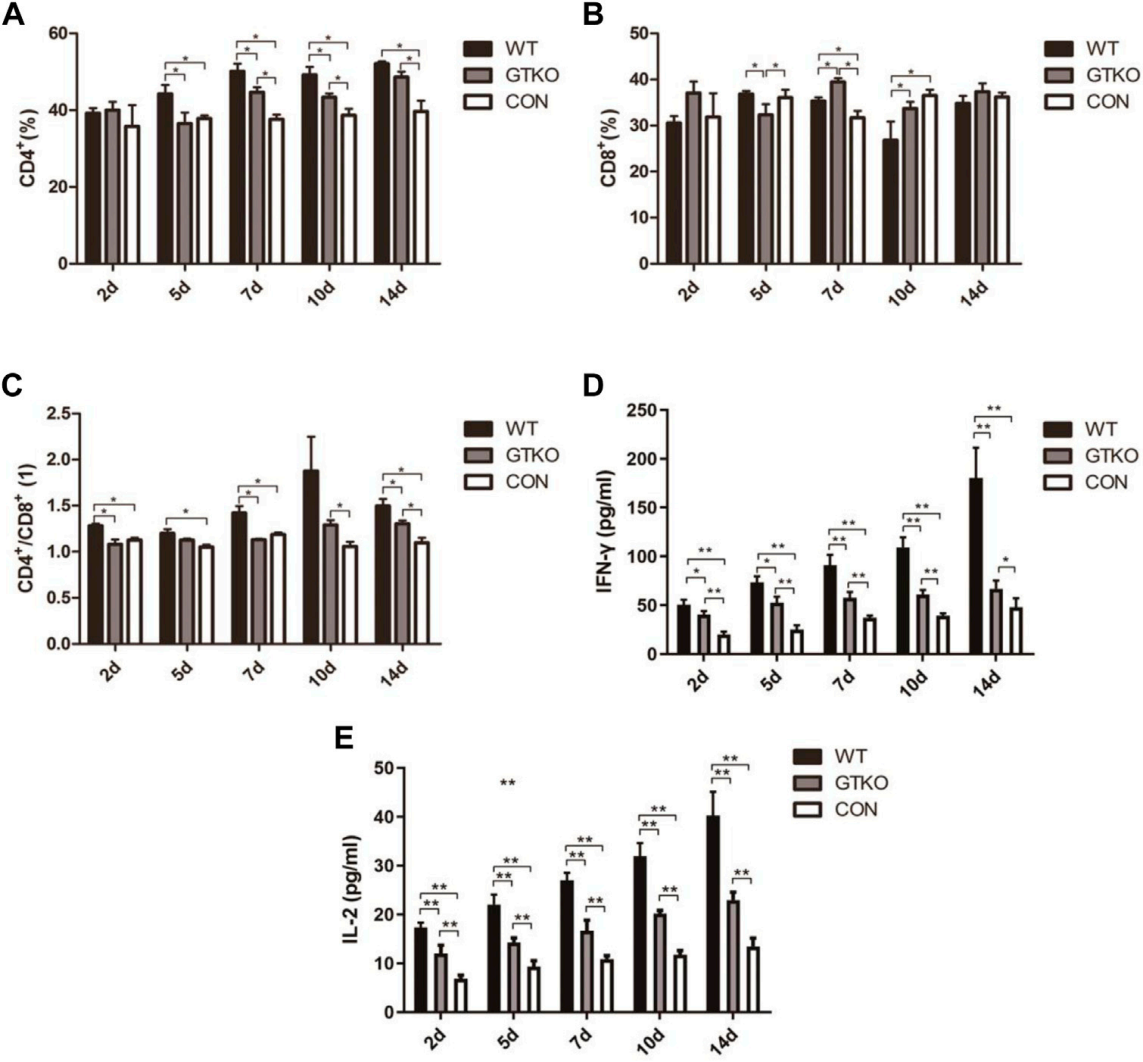
Immunological assessment of xenotransplant rejection. **(A)** Statistical analysis of the percentage of CD4^+^ T lymphocytes in rhesus monkeys transplanted with the materials shown at 2, 5, 7, 10, and 14 days after surgery. WT: defect repaired with WT-pig cancellous bone plug; GTKO: defect repaired with GTKO-pig cancellous bone plug; CON: control group (no treatment); **(B)** Statistical analysis of the percentage of CD8^+^ T lymphocytes in the rhesus monkeys transplanted with the materials shown at 2, 5, 7, 10, and 14 days after surgery; **(C)** Statistical analysis of the ratio of CD4+/CD8+ T lymphocytes in the rhesus monkeys transplanted with the materials shown at 2, 5, 7, 10, and 14 days after surgery. **(D)** Statistical analysis of IFN-γ in the peripheral blood serum of rhesus monkeys transplanted with the materials shown at 2, 5, 7, 10, and 14 days after surgery; **(E)** Statistical analysis of IL-2 from the peripheral blood serum of rhesus monkeys transplanted with the materials shown at 2, 5, 7, 10, and 14 days after surgery. Data are shown as mean ± S.D.*p < 0.05. GTKO, α1,3-galactosyltransferase-knockout pig cancellous bone group; WT, wild type pig cancellous bone group; Con, unrepaired defect group.

To characterize the immunologic responses of rhesus monkeys following xenografting, we analyzed the phenotypic profile of peripheral blood T cells by gating on CD4^+^ or CD8^+^ populations. The CD4^+^ T-cell population of the WT group showed a significant increase at 5, 7, 10 and 14 days post-surgery when compared to the control group (Figure 1A). Although the CD4^+^ T-cell population of the GTKO group also exhibited a marked increase at 7, 10 and 14 days post-surgery (Figure 1A), it was significantly lower compared with the WT group. The CD8^+^ T-cell population of the GTKO group also exhibited a marked increase at 7 and 10 days post-surgery compared with the WT group (Figure 1B).

The most striking difference was that the ratio of CD4^+^ to CD8^+^ T cells of the GTKO group was lower than the WT group at all time points (Figure 1C). However, as compared to the CON group, the GTKO group revealed a significant increase in the ratio of CD4^+^ to CD8^+^ T cells at 10 and 14 days post-surgery.

To further investigate the immunologic response after surgery, the cytokines IFN-γ and IL-2 released in the serum of rhesus monkeys were quantified by sandwich ELISA at 2, 5, 7, 10, and 14 days after operation.

When compared to the negative control (CON), we found that the defects implanted with WT-pig bone and GTKO-pig bone induced a significant elevation in serum IFN-γ at 2, 5, 7 and 10 days post-surgery (Figure 1D). Compared to the GTKO group, IFN-γ concentration was further elevated in the WT group at 5, 7, 10, and 14 days post-surgery (Figure 1D).

Accompanying the IFN-γ response was a significant rise in serum IL-2 in both the WT and the GTKO group, which was likewise elevated at 2, 5, 7, 10, and 14 days post-surgery (Figure 1E). In contrast with the GTKO group, IL-2 concentration was further elevated in the WT group at each time-point post-surgery (Figure 1E). These data indicate that GTKO-pig bone was less xenotransplant rejection than WT-pig bone, which may provide better bone microenvironment.

### Radiographic assessment of new bone formation

**FIGURE 2:**
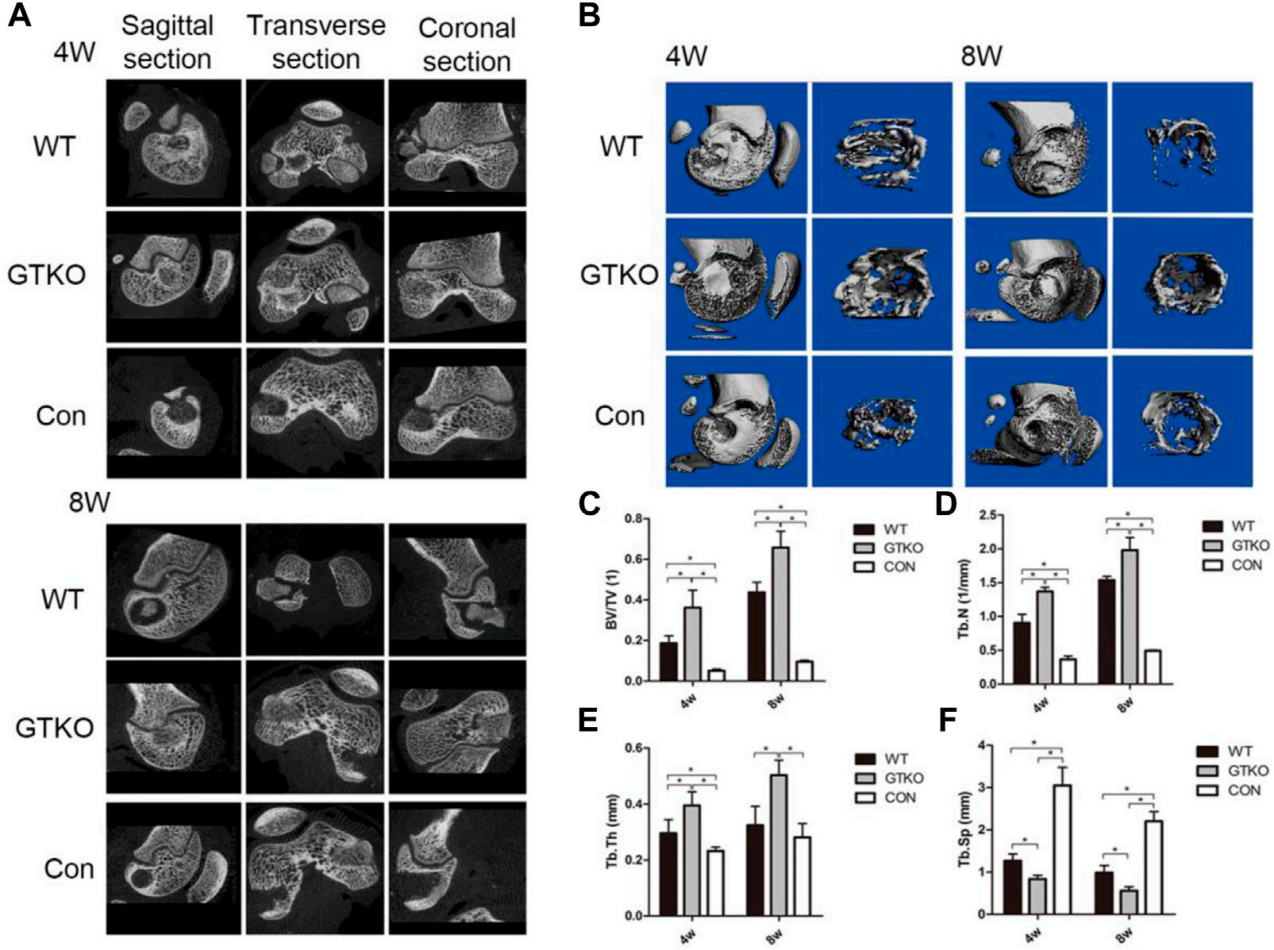
Two-dimensional reconstruction of micro-CT scan. **(A)** Axial, coronal and sagittal reconstruction at 4 and 8 weeks after surgery. **(B)** Three-dimensional reconstruction of a micro-CT scan of the whole femoral condyle and VOI (×2.5) at different time-points after the surgery. **(C)** Statistical analysis of the VOI of the bone defects showing the bone volume fraction by micro-CT at different time-points after surgery; **(D)** Statistical analysis of the VOI of the bone defect showing trabecular number by micro-CT at different time-points after surgery; **(E)** Statistical analysis of the VOI of the bone defect showing trabecular thickness by micro-CT at different time-points after surgery; **(F)** Statistical analysis of the VOI of the bone defect trabecular separation by micro-CT at different time-points after surgery. Data are shown as mean ± SD.*p < 0.05. VOI, volume of interest; BV/TV, trabecular bone volume fraction; Tb. Th, trabecular thickness; Tb. N, trabecular number; Tb. Sp, trabecular separation.

To observe new bone formation within the defects, axial, coronal, and sagittal micro-CT images with two-dimensional reconstruction of the bone tissue around the implants within the defects were collected at 4 and 8 weeks post-surgery (Figure 2A). As can be seen in the untreated defects (CON group), few new bone formation was observed in the defect at either four or 8 weeks after implantation (Figure 2A). Defects in the WT group initially exhibited cylindrical peri-implant radiolucencies around the radiopaque tissue (which was the fresh WT-pig bone graft at 4 weeks; Figure 2A). The radiolucencies surrounding the graft had expanded, while the volume of the bone graft appeared to be reduced in the defects treated with fresh WT-pig bone at 8 weeks (Figure 2B). In contrast, a large filled area of mineralized tissue with few radiolucencies was found in defects filled with fresh GTKO-pig bone graft at 4 weeks post-surgery (Figure 2A). Few radiolucency was noted around the GTKO-pig bone graft at 8 weeks post-surgery, indicating that with extended time, the bone surrounding the defect would tightly integrate with the graft (Figure 2B).

To quantify the mineralized bone formation at the periphery of the implant, micro-CT analysis was conducted using a hollow cylindrical volume of interest (VOI-I) within the defects (Figure 2B). Compared to the negative control (CON), the defects repaired with either WT-pig bone or GTKO-pig bone exhibiting a marked increase in BV/TV, trabecular number and trabecular thickness at 4 and 8 weeks post-surgery (Figures 2C–E), while the trabecular separation in both the WT and GTKO groups were significantly lower than in the CON group (Figure 2F).

The GTKO-pig bone-implanted defects revealed increased mineralized tissue formation when compared to the WT group, as reflected in higher BV/TV, trabecular number, and trabecular thickness (Figures 2C–E) and lower trabecular separation at 4 and 8 weeks (Figure 2F). These data indicate that GTKO-pig bone was more bone formation than WT-pig bone, which may provide good bone conduction ability.

### Histology, immunohistochemistry and histomorphometric assessment

**FIGURE 3:**
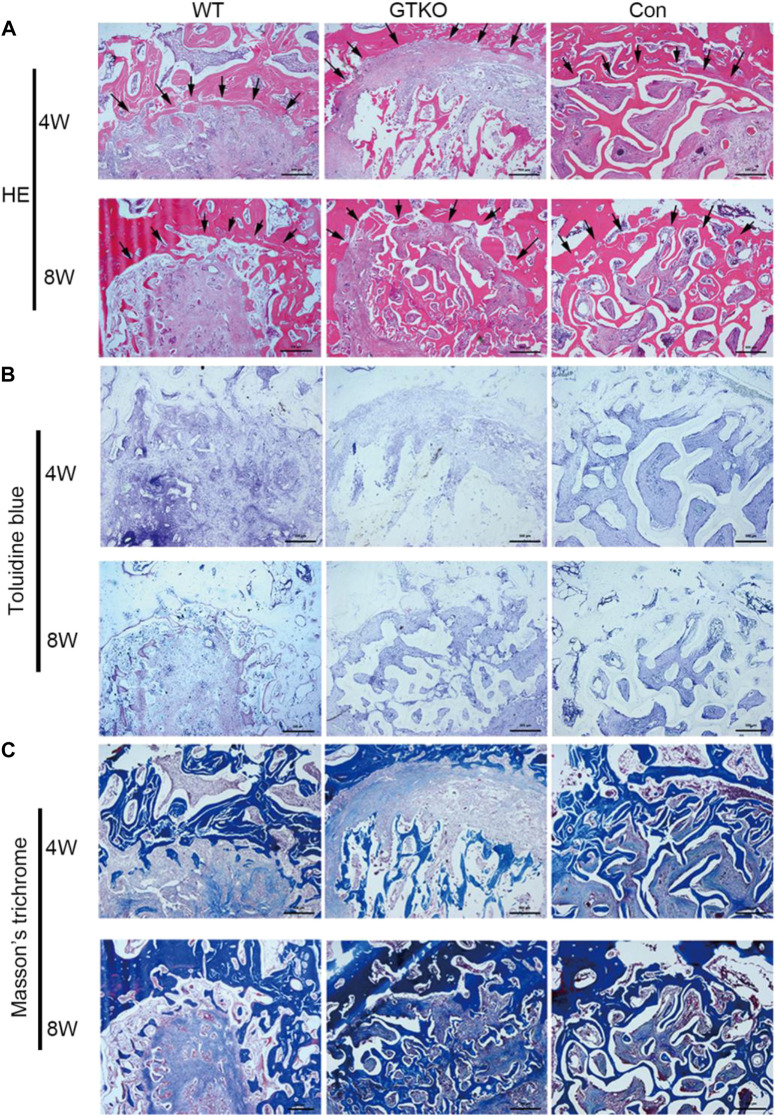
Histochemical assessment. **(A)** HE staining of the bone defect areas transplanted with the materials shown at 4 and 8 weeks after surgery (×25). Arrows indicate the rhesus monkey trabeculae on the edge of the bone defect. **(B)** Toluidine blue staining of the bone defect areas transplanted with the materials shown at 4 and 8 weeks after surgery (×25); **(C)** Masson’s trichrome staining of the bone defect areas transplanted with the specific materials 4 and 8 weeks after surgery (×25). Scale bar, 500 μm.

No obvious inflammation was observed in the defects of any groups. As can be seen in the untreated defects (CON group), abundant fibrous tissue with few blood vessels was observed within the defect at both 4 and 8 weeks postoperatively (Figure 3A). After 8 weeks, cartilage formation and endochondral ossification were observed in some parts of the defect in the CON group (Figure 3B). Although the results of Masson’s trichrome staining suggested that type I collagen fiber accumulation increased over time within the defect, newly-formed bone was observed only on the inner surface of the host bone (Figure 3C).

Defects in the WT group initially demonstrated decreased bone mass of the bone graft and abundant fibrous tissue around the graft, indicating marked bone resorption at 4 weeks postoperatively (Figure 3A), and no graft–host union was present in the WT group.

After 8 weeks, defects treated with WT-pig bone exhibited increased bone mass of the bone graft in contrast with the same group at 4 weeks post-surgery (Figure 3A). In some parts of the surrounding fibrous tissues, accumulation of type I collagen fibers around the graft was increased and newly-formed bone was found to be close to the host bone (Figure 3C). Nevertheless, no graft–host union was present within the defects of the WT group. When compared with the WT group, defects of the GTKO group demonstrated a notable increase in bone mass of the bone graft at 8 weeks post-surgery (Figure 3A). The newly-formed bone within the defects of the GTKO group grew into the spaces between the graft trabeculae and connected directly with the host bone (Figures 3A,C).

**FIGURE 4:**
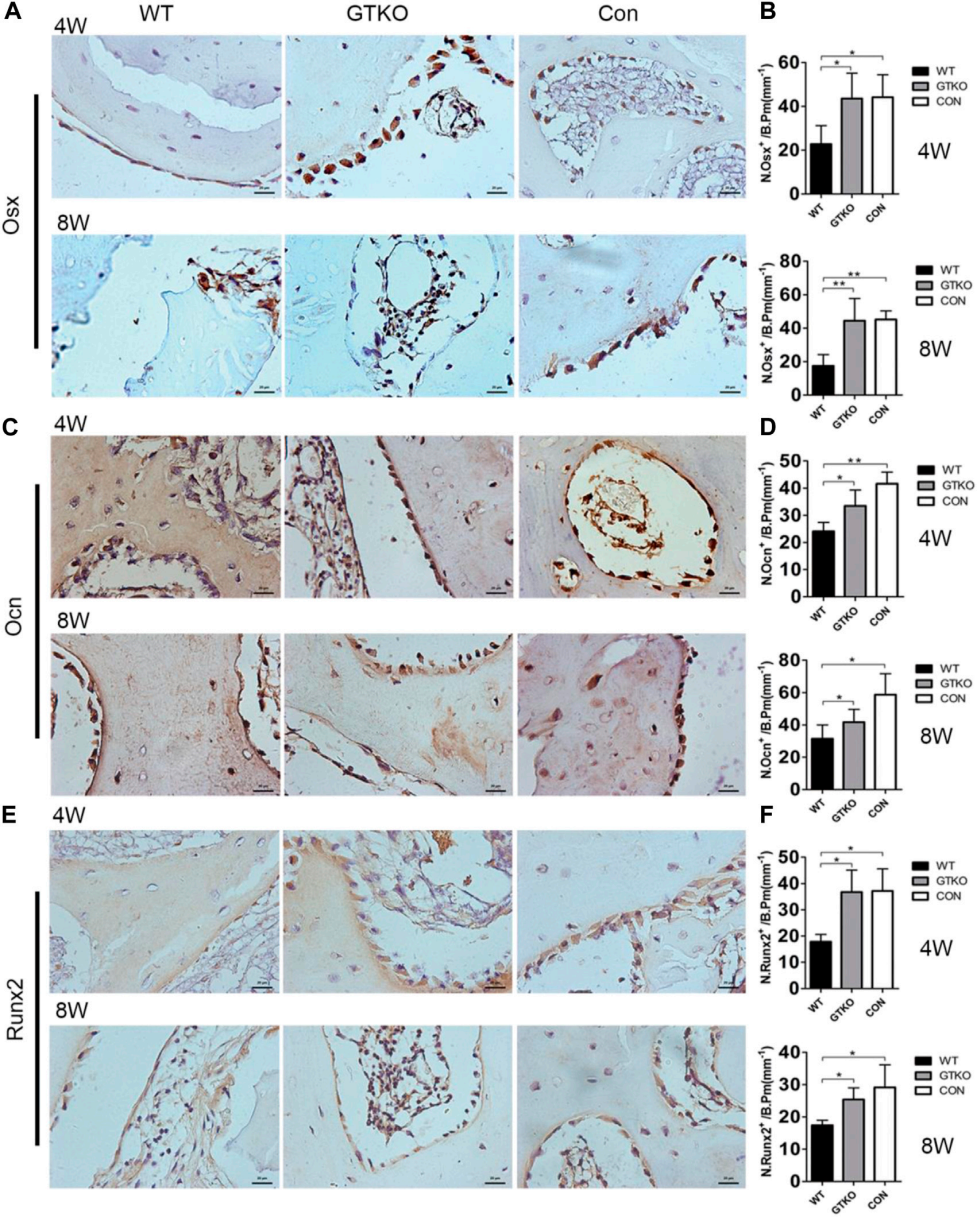
Immunohistochemical staining of markers of osteoblast differentiation. **(A,B)** OSX immunohistochemical staining of the bone defect areas transplanted with the materials shown, 4 and 8 weeks after surgery (×400) and statistical analysis of the number of OSX-positive cells on the bone perimeter (N. Osx+/B Pm); **(C,D)** OCN immunohistochemical staining of the bone defect areas transplanted with the materials shown, 4 and 8 weeks after surgery (×400) and statistical analysis of the number of osteoblasts on the bone perimeter (N. Ocn+/B Pm); **(E,F)** Runx2 immunohistochemical staining of the bone defect areas transplanted with the materials shown, 4 weeks after surgery (×400) and statistical analysis of the number of Runx2-positive cells on the bone perimeter (N. Runx2+/B Pm). Scale bar, 20 μm. Data are shown as mean ± SD. *p < 0.05, **p < 0.01. Osx, osterix; Ocn, osteocalcin; Runx2, runt-related transcription factor 2.

To determine whether the number and differentiation of osteoblasts were affected, their numbers at different stages of differentiation were measured by immunohistochemical staining of sections for markers of osteoblast differentiation. The number of OSX-positive preosteoblasts (Figures 4A,B) and OCN-positive mature osteoblasts (Figures 4C,D) on the bone surfaces of the GTKO group were significantly higher than those in the WT group. However, no significant differences in the numbers of OSX-positive preosteoblasts or OCN-positive mature osteoblasts were found between the CON group and the GTKO group (Figures 4A–D). The differentiation of osteoblasts was further confirmed in sections from each group by immunohistochemical staining for Runx2, a major transcription factor which is required for commitment of mesenchymal osteochondroprogenitors to the osteoblastic lineage, differentiation into mature osteoblasts and terminal differentiation into osteocytes (Lin and Hankenson, 2011). The defects repaired with GTKO-pig bone exhibited a marked increase in Runx2 expression when compared with the WT group (Figures 4E,F).

**FIGURE 5:**
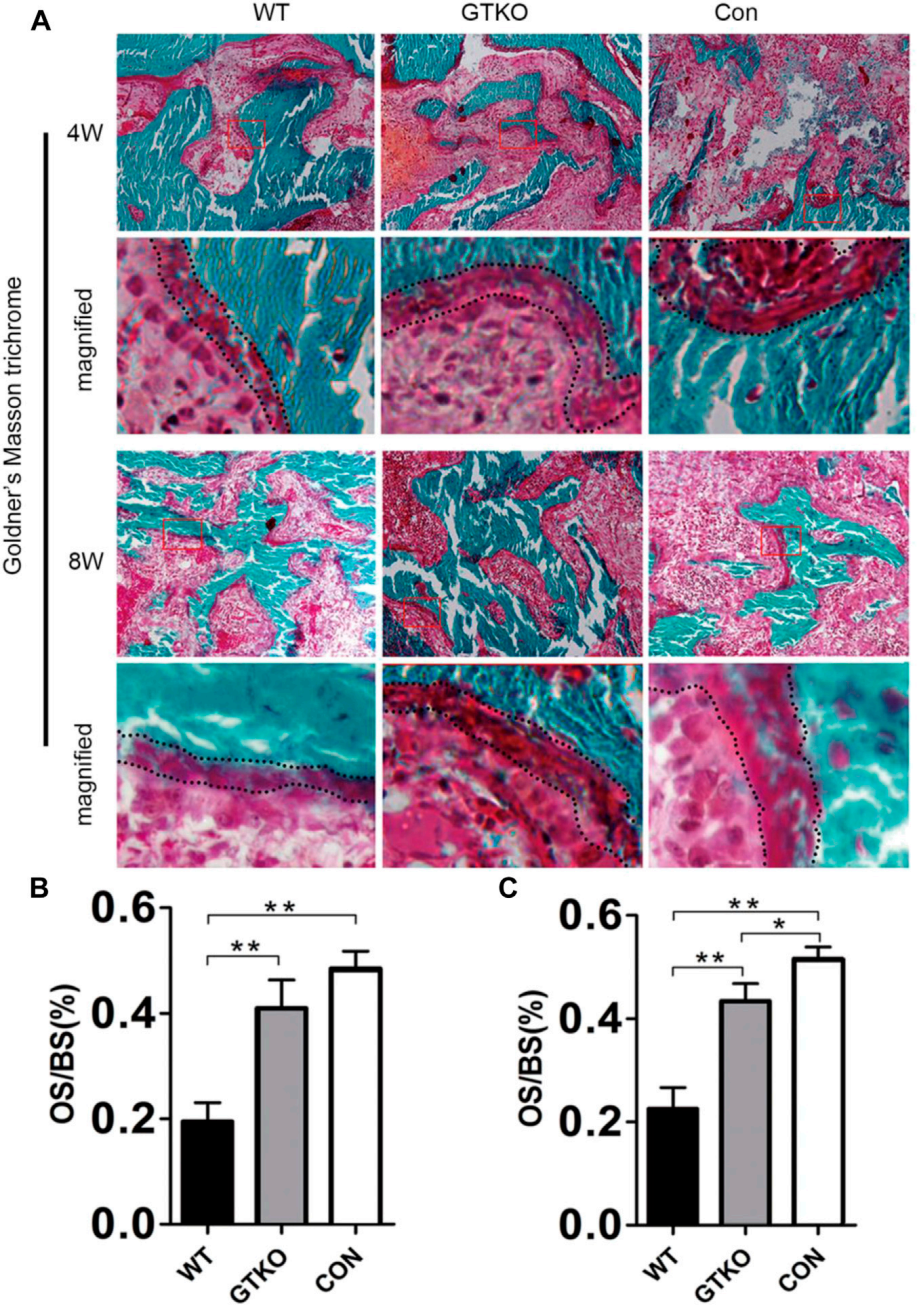
Histomorphometric assessment of new bone regeneration. **(A)**: Goldner’s Masson trichrome staining of the bone defect areas transplanted with the materials shown, 4 and 8 weeks after surgery (×200) and **(B,C)** statistical analysis of the osteoid per bone surface (OS/BS). Boxed area is enlarged in the panel below; the osteoid border is marked by a dotted line. Data are shown as mean ± SD.*p < 0.05, **p < 0.01.

The results of Goldner’s Masson trichrome staining in undecalcified histology sections indicated that the defects of the GTKO group had 21.5% and 20.9% more osteoid/hypomineralized areas (stained red) in bone than the WT group at 4 and 8 weeks, respectively (Figures 5A–C). No significant difference was found between the GTKO group and the CON group at 4 weeks (Figure 5B). Together, these findings, which were in line with results from immunohistochemical staining of osteoblastic markers (OSX and OCN) revealed that GTKO-pig bone induced a marked increase in new bone regeneration.

## Discussion

In this study, GTKO-pig cancellous bone was transplanted into cylindrical bone defects (diameter 5 mm, depth 8 mm) of rhesus monkeys to evaluate its bone healing effect andinfluences on heterologous immune rejection. The results of micro-CT and histological staining of the control groups at either four or 8 weeks post-surgery indicated that the rhesus monkey cylindrical bone defect model is up to critical size defect (CSD) standard (Hollinger and Kleinschmidt, 1990). At present, the most commonly-used bone defect models are the segmental bone defect and cylindrical bone defect (Xie et al., 2007). However, rigid external or internal fixation is absolutely essential in the monkey segmental bone defect model because of the high activity level of rhesus monkeys. Meanwhile the cylindrical bone defect model can provide appropriate long-term stability and biomechanistic environments without any external or internal fixation. A recent study showed that alpha-galactosidase treatment of a porcine xenobone graft can reduce the alpha-gal epitope and present better bone healing by reduce the humoral immune response to the alpha-gal antigen in C57/BL6 alpha-gal knockout mice (Park et al., 2014). However, few studies have been carried out into GTKO-pig bone xenotransplantation in non-human primate models. As the bone microstructure of the monkey is more similar to swine, the cylindrical bone defect model in rhesus monkeys is a more scientifically-sound evaluation model for detecting the effects of alpha-Gal epitope knockout on bone healing. Thus, our findings suggest that GTKO-pig cancellous bone showed better bone healing by immunological assessment and radiographic assessment (Scheme 1). The combination of immunological analysis and radiographic analysis is an effective method for bone healing.

In consideration of their ease of breeding and feeding, their similarity to humans in size, and the relatively low cost of artificial propagation, pigs are the most appropriate xenograft animal donor for humans. However, α-1,3-Gal epitopes are the major xenoantigens resulting in hyperacute rejection (HAR) in pig-to-human xenotransplantation (Cooper et al., 1993). Alpha-Gal expression has been observed on the surface of osteocytes and in Haversian canals; however, it is not expressed in the extracellular matrix of bone (Feng et al., 2006). Moreover, alpha-Gal expression on the surface of bone microvascular endothelial cells would result in both hyperacute and chronic immune rejection, which are mediated by T lymphocytes in heterologous bone transplantation (Yu et al., 2015). The α1,3-GT gene knockout (GTKO) pigs have made pig-to-human xenotransplantation possible. Hering et al. (2006) confirmed that suppressed CD4^+^ T cells would prolong the graft survival time. The result of flow cytometric analysis of the peripheral blood T cells’ phenotypic profile showed that knockout of the α1,3-GT gene would reduce the percentage of CD4^+^ T lymphocytes as well as the ratio of CD4^+^ and CD8^+^ T lymphocytes. IL-2 and INF-γ, which promote T lymphocyte activation and proliferation to promote immunological rejection, are confirmed to be secreted by CD4^+^ T lymphocytes (Wan and Flavell, 2009; Hermann-Kleiter and Baier, 2010). We found that the expression of IL-2 and INF-γ in the GTKO group were both relatively lower than in the WT group, which proved that immunological rejection could be reduced by α1,3-GT gene knockout. Nevertheless, immunological rejection in the GTKO group remained higher than in the CON group. These data would suggest that non-Gal epitopes could also cause xenotransplant rejection.

Radiological and histological examinations were performed to evaluate the osteoconductive and osteoinductive properties of GTKO-pig bone. The three-dimensional (3D) reconstructed micro-CT images showed no fibrous capsule formation, whereas there was abundant new bone formation surrounding the GTKO bone graft. Moreover, the newly-formed bone within the defects of the GTKO group grew into the spaces between the graft trabeculae and connected directly with the host bone, showing good osseointegration. These results suggested that GTKO-pig bone was more osteoconductive than WT pig bone. These images also showed that the bone resorption rate and chronic inflammatory response could be alleviated by α1,3-GT gene knockout. The above results suggest that GTKO might be osteoinductive and biocompatible. Osteoblasts, which are the main bone-forming cells, differentiate and produce bone matrix to build new bone (Ducy et al., 2000). The osteoblast phenotype progression is often divided into stages of mesenchymal progenitors, preosteoblasts and osteoblasts (often called mature osteoblasts) (Long, 2011). Osteoblasts are often characterized by the expression of OCN, while preosteoblasts are usually considered to express the transcription factor Runx2 or both Runx2 and OSX (Huang et al., 2015). The osteoinductivity of GTKO-pig bone was further confirmed by immunohistochemical staining of mesenchymal progenitors, preosteoblasts and osteoblasts. These results suggested that the osteoblast phenotype progression in the GTKO group was not affected by GTKO when compared with the CON group. Moreover, images of Goldner’s Masson trichrome staining showed that GTKO enhanced the osteoid formation of pig bone grafts. Due to a reduced number of OSX- and OCN-positive cells, only a small amount of osteoid was observed around the WT-pig bone graft. These results suggest that GTKO-pig bone might have good osteoinductivity. Furthermore, these data concerning the inductive function of the graft bone also proved that GTKO could reduce xenotransplant rejection. However, in order to minimize the surgical incision, sometimes the cylindrical bone defect passed through the growth plate. It would be a limitation as the bone formation differs between growth plate and trabecular bone. Clinically, chronic rejection may still occur 2–3 years after transplantation and this study should be prolonged observation (24 and 48 weeks). Within the limitations of the proposed research, the present study focused on investigating the influence on immunological rejection, osteoconductivity and osteoinductivity of GTKO for orthopedic applications, but future mechanistic studies for modifying GTKO-pig bone to provide an ideal bone graft substitute are still warranted.

Tissue engineering is an innovative technology, which as an alternative strategy to treat damaged organs and tissues (Thavornyutikarn et al., 2014). An ideal scaffold for bone tissue engineering can provide biocompatibility, osteoconductivity and biodegradability. However, the residual solvents and porogens left in the scaffold material could denature proteins, and thus be harmful to cells and biological tissues. And GTKO-pig bone, which don’t exist solvent toxicity, can inhibit xenotransplant rejection provide osteoconductivity. Moreover, the biomaterial replacement technique is a good treatment for massive bone defect. The biomaterial replacement technique can provide specific bone models, such as humeral head, femoral head and acetabulum (Pruksakorn et al., 2015). While GTKO-pig bone only provide diaphysis bone repair. Together, xenogeneic bone combined with bone tissue engineering and biomaterial replacement technique may be a good strategy.

In this study, as a new source of material for xenotransplantation, GTKO-pig cancellous bone showed good biocompatibility, good osteoconductivity and relatively low immune rejection. Compared with WT-pig bone, the heterologous immunogenicity of GTKO-pig bone was, if not eliminated, at least relatively reduced. From the histological prospect, GTKO-pig cancellous bone showed better osteoinductivity suggesting it could be able to promote and accelerate the bone healing process. An appropriate rate of absorption made it possible for GTKO-pig bone to fill and maintain the structure of bone defects. Although GTKO-pig cancellous bone still has a certain degree of heterologous immunogenicity, the results in our study illustrated that α1,3-GT knockout makes a positive contribution to reducing immune rejection. Therefore, further study into the mechanism of heterologous immune rejection induced by non-gal antigens as well as new genetically-modified transgenic pigs will realize the great potential of GTKO-pig cancellous bone for clinical application. According to the results of this study, new ideas and some experimental bases are provided for bone defect repair material development and innovation.

## Conclusion

GTKO-pig cancellous bone showed better bone healing by inhibiting xenotransplant rejection and promoting new bone formation. GTKO group decreased the ratio of CD4^+^ to CD8^+^ T cells and cytokines (IFN-γ and IL-2) to inhibit xenotransplant rejection. Moreover, GTKO group increased more bone formation by micro-CT analysis and osteoblastic markers (Runx2, OSX and OCN). Together, GTKO-pig cancellous bone showed better bone repair than WT-pig cancellous bone.

## Data Availability

The original contributions presented in the study are included in the article/Supplementary Material, further inquiries can be directed to the corresponding authors.
