# Supplementary material for: Repair of bone defects in rhesus monkeys with α1,3-galactosyltransferase-knockout pig cancellous bone
Source: Front Bioeng Biotechnol. 2022 Sep 12;10:990769. doi: 10.3389/fbioe.2022.990769 (PMC9510634; doi:10.3389/fbioe.2022.990769)
Supplement: Supplementary file 1 [file DataSheet1.doc]

**Supplementary file**


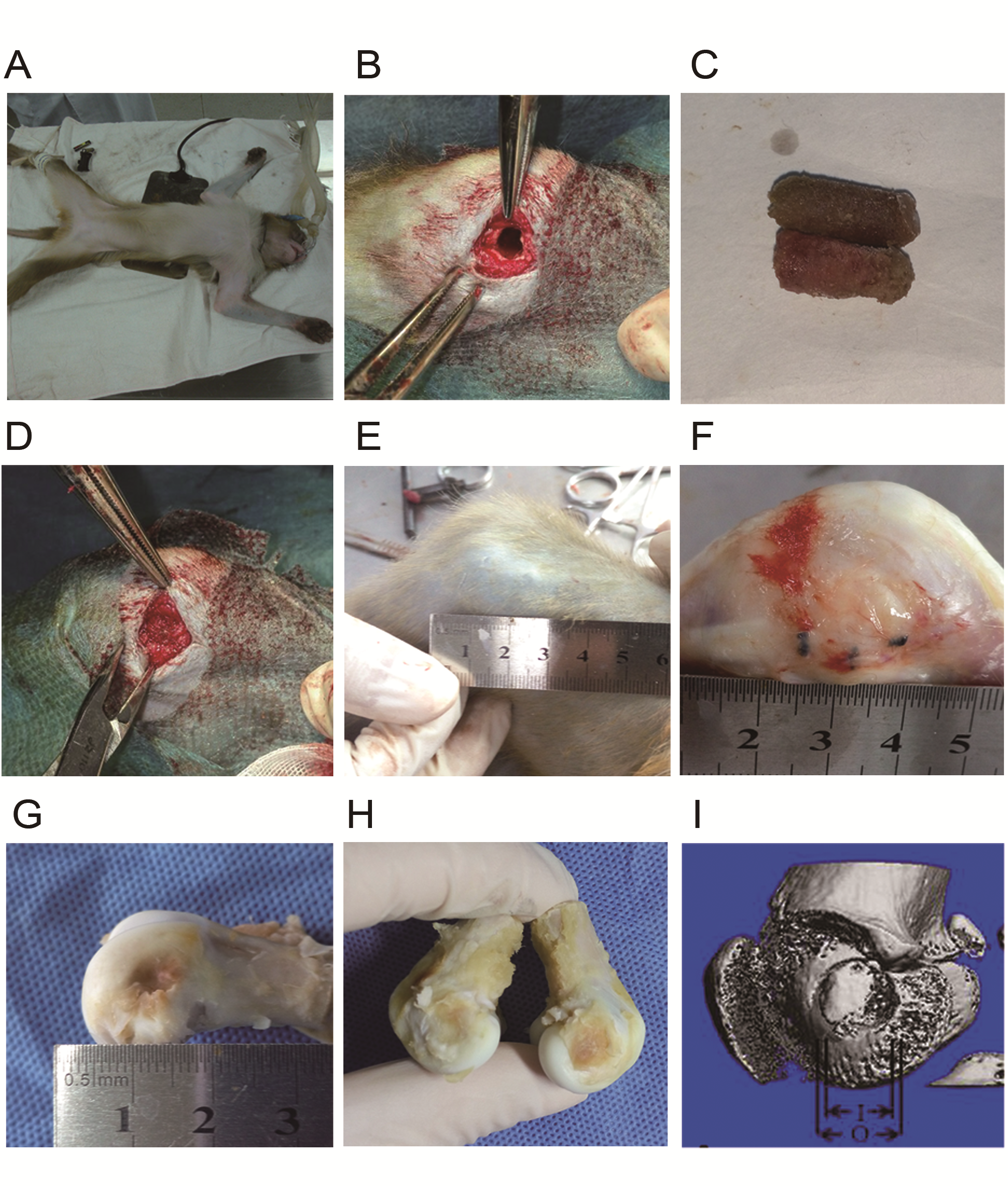


**Supplementary figure. 1.** Diagrammatic illustration of rhesus monkey femoral bone hole defect surgery: A: Endotracheal intubation and inhalation anesthesia; B: Cylindrical bone defect (diameter 5 mm, depth 8 mm) was created with a mosaic plasty harvester; C: WT-pig cancellous bone plug (upper), GTKO-pig cancellous bone plug (lower); D: Implants matching the size of the defect were inserted via press fit; E: The wounds in the rhesus monkeys healed well, with no obvious local swelling, exudate, or other postoperative complications such as sinus formation; F: Monkeys were anesthetized and sacrificed; G: Gross appearance of representative CON group specimen; H: Gross appearance of GTKO group (left) and WT group ( right) specimens; I: The Volume of Interest (VOI). O: Outer circle (5.0 mm), I: Inner circle (4.0 mm).
